# Tyrosine Kinase ROR1 as a Target for Anti-Cancer Therapies

**DOI:** 10.3389/fonc.2021.680834

**Published:** 2021-05-28

**Authors:** Yuming Zhao, Dengyang Zhang, Yao Guo, Bo Lu, Zhizhuang Joe Zhao, Xiaojun Xu, Yun Chen

**Affiliations:** ^1^ Edmond H. Fischer Translational Medical Research Laboratory, Scientific Research Center, The Seventh Affiliated Hospital, Sun Yat-Sen University, Shenzhen, China; ^2^ Department of Hematology, The Seventh Affiliated Hospital, Sun Yat-Sen University, Shenzhen, China; ^3^ Department of Pathology, University of Oklahoma Health Sciences Center, Oklahoma City, OK, United States

**Keywords:** ROR1, kinase activity, kinase function, small molecule inhibitors, targeted therapy

## Abstract

Receptor tyrosine kinase ROR1 plays an essential role in embryogenesis and is overexpressed in many types of malignant tumors. Studies have demonstrated that it plays an important role in oncogenesis by activating cell survival signaling events, particularly the non-canonical WNT signaling pathway. Antibody-based immunotherapies targeting ROR1 have been developed and evaluated in preclinical and clinical studies with promising outcomes. However, small molecule inhibitors targeting ROR1 are underappreciated because of the initial characterization of ROR1 as a peusdokinase. The function of ROR1 as a tyrosine kinase remains poorly understood, although accumulating evidence have demonstrated its intrinsic tyrosine kinase activity. In this review, we analyzed the structural and functional features of ROR1 and discussed therapeutic strategies targeting this kinase.

## Introduction

Receptor tyrosine kinases (RTKs) are key regulators of normal cellular processes, and they are also involved in the development and progression of many types of cancer (1). To date, the RTKs superfamily has 58 members that fall into 20 subfamilies. The receptor tyrosine kinase-like orphan receptor (ROR) subfamily contains two members, namely, ROR1 and ROR2 that were initially identified in a human neuroblastoma cell line in 1992 (2). ROR1 and ROR2 are evolutionarily conserved among animals including protostomes, cnidarians, and all vertebrates, suggesting a critical physiological function of ROR family members in development. The ROR subfamily members were designated as “orphan” receptors because their ligands have not been identified for many years. Now it is known that ROR family members are receptors for Wnt family signaling molecules Wnt5a/b and Wnt16, with Wnt5a as the primary ligand (3–6).

The Wnt family of proteins transduce signals through the canonical β-catenin-dependent and non-canonical β-catenin-independent pathways (7, 8). Several Wnt ligands, including Wnt1, Wnt3a, and Wnt8, can bind to the Frizzled family of receptors to trigger the β-catenin-dependent Wnt signaling pathway. This activated signaling cascade stabilizes β-catenin to facilitate transcription of pro-survival genes, including Myc, Survivin, and MMP (9–11). In contrast, other Wnt ligands such as Wnt5a/b and Wnt16 bind to the ROR family proteins and activate β-catenin-independent Wnt/planar cell polarity and Wnt/Ca^2+^ pathways, involving activation of small GTPases Rho/Rac signaling proteins and the protein kinase C (PKC)/calcineurin signaling cascade, respectively. Although insufficiently studied, Wnt β-catenin-independent pathways are known to regulate cell polarity, proliferation, motility, and migration (12, 13). Studies have shown that binding of Wnt5a leads to autophosphorylation of ROR2, which induces activation of β-Catenin-independent pathways (14). However, autophosphorylation of ROR1 and the associated downstream signaling network are still poorly understood.

**Figure 1:**
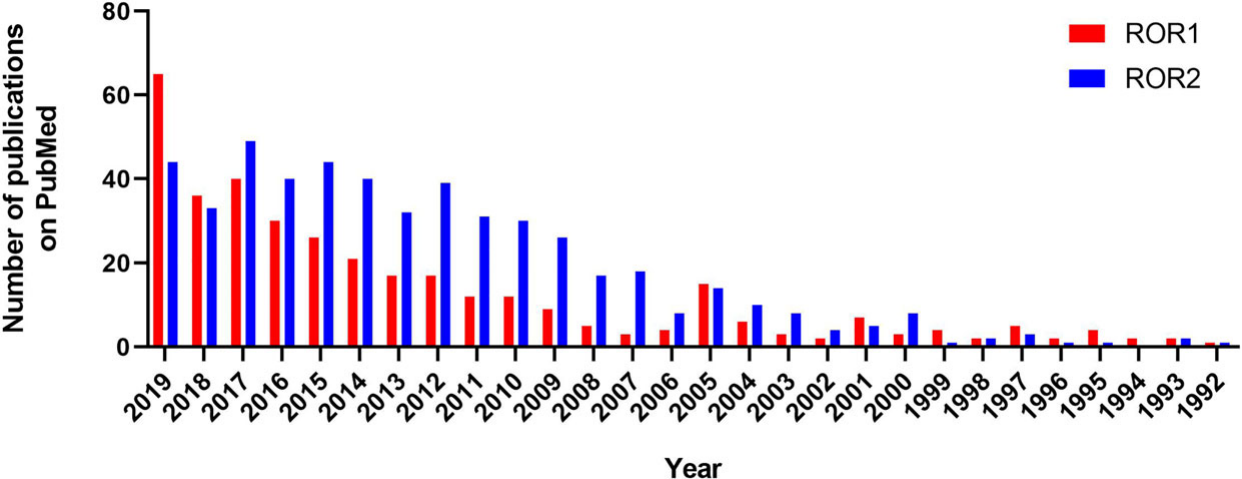
Numbers of publications on ROR1 and ROR2 between 1992 to 2019 according to PubMed.

Until recently, ROR2 has been studied more intensively than ROR1 (**Figure 1**). This was due to a clear association of ROR2 mutations with two distinct developmental defects in humans, namely, Robinow syndrome and type B1 brachydactyly (15). Although no mutations in ROR1 have been found in any human disease yet, numerous studies demonstrated aberrant expression patterns of ROR1 in many types of diseases in recent years, which made ROR1 an attractive therapeutic target in malignancies, ischemia, and diabetes (16–18), leading to a growing interest in studying ROR1 signaling and targeted therapies against ROR1 (15).

This review provides updated information about the structural and functional features of ROR1, its role in cell signaling, and anti-cancer therapies targeting ROR1. It further discusses the potential of ROR1 as an active tyrosine kinase and strategies to identify small-molecule compounds that inhibit ROR1 signaling.

## The Structure and Expression Pattern of ROR1

**Figure 2:**
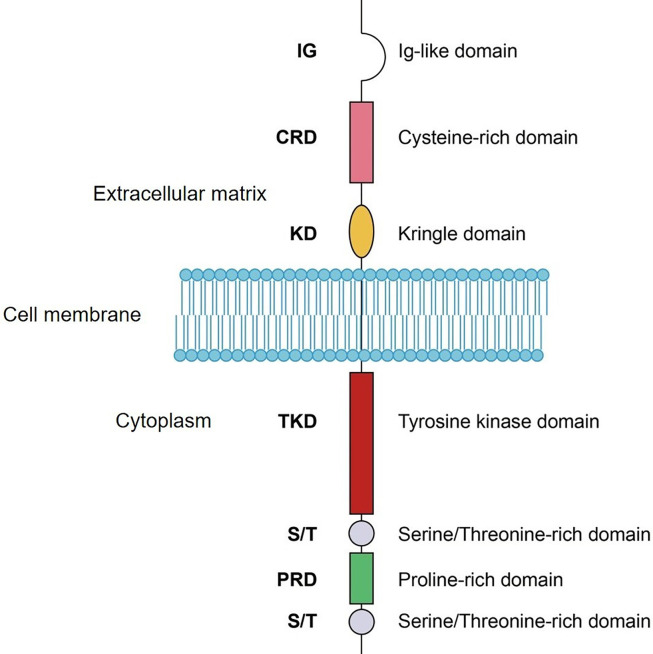
Schematic domain structure of ROR1. IG, Ig-like domain; CRD, cysteine-rich domain; KD, kringle domain; TKD, tyrosine kinase domain; S/T, serine/Threonine-rich domain; PRD, proline-rich domain.

ROR1 is a transmembrane receptor that contains an extracellular section, a transmembrane segment, and a cytoplasmic region (**Figure 2**) (2). The extracellular part comprises an immunoglobulin-like domain (IG), a cysteine rich domain (CRD), and a Kringle domain (KD). CRD modulates non-canonical WNT signaling by binding to the ligand Wnt5a (19, 20). KD mediates the interaction of ROR1 with other receptors such as ROR2 (21). The cytoplasmic section includes a tyrosine kinase domain, two serine/threonine-rich domains, and a proline-rich domain. The proline-rich domain is responsible for the activation of cell migration and proliferation signals by recruiting SH3 domain-containing proteins including HS1, DOCK2, and cortactin (22–24). The serine/threonine-rich domain physically interacts with adaptor proteins, such as 14-3-3 zeta, leading to resistance to apoptosis (25). The detailed signaling activated by ROR1 has been reviewed recently (7). Although ROR1 is a member of the receptor tyrosine kinase superfamily, the function of the tyrosine kinase domain remains controversial.

The gene encoding ROR1 was first identified in a human neuroblastoma cell line in 1992 (2), and subsequent studies found that ROR1 was expressed predominantly during embryogenesis, mainly in the central nervous system, the early limb bud, cartilage condensations, cartilage growth plate, heart, lung, and mesonephros in mice (15). ROR1 knockout is embryonic lethal, indicating a critical role of ROR1 in embryogenesis (26). ROR1 is not expressed in most postpartum tissues, except for adipose tissues that highly express ROR1, and some tissues from the endocrine glands, gastrointestinal tract, and immature B lymphocytes (27, 28). The function of ROR1 in these adult tissues is currently unknown.

## The Role of ROR1 in Malignancies

Many studies have shown that ROR1 was expressed at high levels in malignant cells. It was first identified as an oncoembryonic gene in hematological malignancies. In 2001, two groups observed high transcriptional levels of ROR1 in chronic lymphocytic leukemia (CLL) cells compared with those in normal B lymphocytes by cDNA microarrays (29, 30). However, the importance of ROR1 was not recognized until years later. In 2008, Fukuda et al. identified aberrantly expressed ROR1 in CLL cells at the protein level using monoclonal antibodies (mAbs). This study also identified Wnt5a as the ligand of ROR1, which stimulated activation of pro-survival signals in a ROR1-dependent manner (31). The expression of ROR1 was also found to have a significant prognostic impact in patients with CLL (32). In addition, ROR1 was found expressed in a subgroup of B acute lymphoblastic leukemia (B-ALL), mainly in patients with t(1;19) chromosomal translocation (33). These B-ALL cells are sensitive to siRNA-mediated ROR1 silencing, indicating a critical function of ROR1 to maintain leukemia-cell survival (34). ROR1 is also aberrantly expressed in mantle cell lymphoma (MCL), triggering pro-survival signals similar to those in CLL and B-ALL (35, 36). Studies found that several other types of non-Hodgkin lymphoma (NHL), including diffuse large B cell lymphoma (DLBCL), follicular lymphoma, and marginal zone lymphoma expressed ROR1, but its function was not clearly characterized (37, 38).

In addition, ROR1 was found in CD34^+^ acute myeloid leukemia (AML) cells and can be targeted by mAbs (39).In addition to hematological malignancies, aberrant expression of ROR1 was also found in a wide range of solid tumors as a biomarker and therapeutic target (7, 40). In breast cancer, ROR1 was found to be positive in tumor specimens but not normal breast tissues (41). High expression of ROR1 in breast adenocarcinoma was associated with epithelial-mesenchymal transition (EMT), tumor metastasis, and aggressive disease (41–43). ROR1 was also found at high levels in lung cancer cells, which serves as a prognostic biomarker in patients with lung adenocarcinoma. Silencing of ROR1 led to growth inhibition in cell lines representing human lung adenocarcinoma (44, 45). In ovarian cancer, tumor cells with high expression of ROR1 exhibited stem cell-like gene-expression signatures and had a greater capacity to engraft immunodeficient mice (46). There are also reports on the expression of ROR1 in other types of cancer, including colorectal cancer, endometrial cancer, gastric cancer, melanoma, and pancreatic cancer (47–51).

ROR1 is not only aberrantly expressed in malignant cells, but is also involved in the activation of signaling proteins important for cell proliferation, survival, and migration. In CLL, Wnt5a/ROR1 activates Rho-GTPase RhoA and Rac1 through the ARHGEF family adaptor proteins, including ARHGEF1, ARHGEF2, and ARHGEF6 (21). Wnt5a also induces tyrosine phosphorylation of HS1, leading to a ROR1-dependent cell migration (22). It is possible that HS1 is directly phosphorylated by ROR1, but more definitive evidence for this possibility is needed. In addition, Wnt5a/ROR1 induces activation of the NF-κB signaling pathway, resulting in autocrine regulation of pro-inflammatory cytokines such as IL-6 (4). Expression levels of ROR1 are tightly correlated with the progression of CLL, making it a good biomarker for prognosis (32). In lung cancer, ROR1 is involved in the activation of c-Src and MET, causing inhibition of tumor cell apoptosis (44, 52). However, these studies did not define whether ROR1 actively phosphorylated c-Src and MET or merely served as their substrate. ROR1 was also found as a scaffold protein of cavin-1 and caveolin-1, which activated AKT in lung adenocarcinoma (53). More systematic investigations are required to validate the precise role of ROR1 in lung cancer cells. In breast cancer, ROR1 promoted activation of the PI3K/AKT pathway, and high ROR1 expression was correlated with more severe progression of the disease (41, 54). Together, aberrant expression of ROR1 and associated pro-growth signaling events are observed in many types of malignancies, making ROR1 an attractive therapeutic target for anti-cancer drug development.

## The Kinase Activity of ROR1

ROR1 and ROR2 were first cloned in 1992 by Dr. Piotr Masiakowski and Dr. Robert D. Carroll. The study found that ROR1 and ROR2 carry the YXXXYY amino acid sequence motif, corresponding to the autophosphorylation site of the insulin receptor. The kinase assay with radiolabeled [γ (32)P]ATP and recombinant proteins carrying tyrosine kinase domains from ROR1 or ROR2 purified from COS cells indicated clear intrinsic kinase activity of ROR2 but weak activity of ROR1 (2). The data about kinase activity of ROR1 are difficult to interpret due to possible contamination by other residual kinases co-purified within the reaction system, while the intrinsic tyrosine kinase activity of ROR2 was repeatedly confirmed by other groups (14, 55–57). Following the initial study, Gentile et al. investigated the kinase activity of ROR1 by using a similar method (52). The kinase assay showed that the autocatalytic activity of ROR1 purified from COS-7 cells was negligible, compared with the classic receptor tyrosine kinase ErbB2. In addition, purified ROR1 failed to phosphorylate the exogenous peptide substrate, and COS-7 cells overexpressing ROR1 did not exhibit elevated autophosphorylation or a change in the tyrosine phosphorylation pattern of endogenous proteins (58). Travis W. Bainbridge and colleagues also found that recombinant proteins carrying the intracellular domain of ROR1 purified from insect cells lacked robust kinase activity in a kinase assay with [γ32P]ATP, largely due to substitutions of several highly conserved amino acids in a GXGXXG motif in the kinase domain compared with other active tyrosine kinases (59). This notion is supported by a recent structural analysis showing that ROR1 has an inaccessible ATP-binding pocket and maintains inactive conformation in the activation loop, because of substitutions of several conserved amino acids. However, the kinase dead mutation (K506A) and autophosphorylation site mutation (YXXXYY to FXXXFF) in ROR1 abolished the ability of ROR1 to support BaF3 cell proliferation, suggesting important signaling functions of the kinase domain of ROR1, even without the ATP binding capability (60).

In contrast, accumulating evidence suggests that ROR1 has intrinsic kinase activity. The group of Dr. Håkan Mellstedt found constitutive phosphorylation of ROR1 in CLL cells, which was associated with progressive disease. Binding of mAbs to the extracellular domains of ROR1 led to rapid dephosphorylation before leukemia-cell apoptosis. The detailed mechanism requires further investigation, but one possibility mentioned in this study is that ROR1 phosphorylation could be ligand-dependent and binding of the mAbs may have prevented binding of the ligand (61). Indeed, this work led to the development of two small molecule inhibitors of ROR1 by the same group, as described in the next section (62–64). The group led by Dr. Takashi Takahashi investigated the role of ROR1 in lung adenocarcinoma in a series of studies and found that ROR1 supported pro-survival signaling in both kinase-dependent and kinase-independent manner. The kinase activity of ROR1 was shown to be required for the regulation of HIF-1α expression (65), repression of the ASK1-p38 axis, oxidative stress-induced cell death (66), and phosphorylation of c-Src (44). On the other hand, ROR1 can serve as a scaffold protein in a kinase-independent manner to facilitate cavin-1/caveolin-1 interaction (53, 67) and binding to HSP90α (67). In another study by Li et al., ROR1 was shown to phosphorylate HER3 thereby mediating bone metastasis of breast cancer *via* crosstalking with the Hippo-YAP pathway (68).

**Figure 3:**
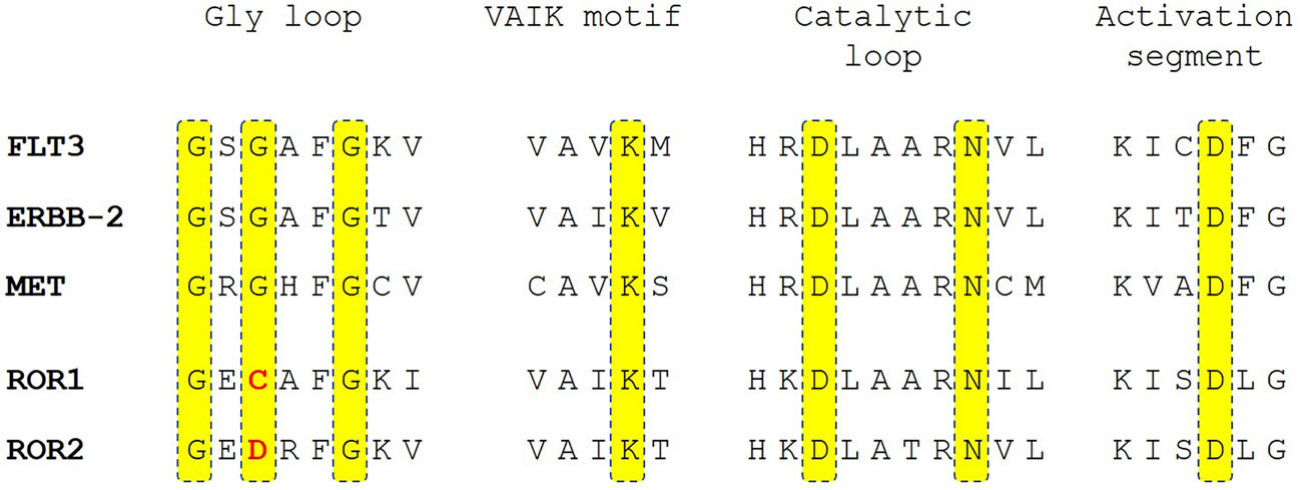
Sequence alignment of ROR1 and ROR2 with active receptor tyrosine kinase receptors FLT3, ERBB-2, and MET in the key peptide segments of protein kinase domains. Highlighted in yellow are key residues involved in enzymatic activity in protein kinases. The deviations of ROR1 and ROR2 sequence from the consensus sequence are highlighted in red.

We believe that ROR1 has an intrinsic tyrosine kinase activity. Protein sequence alignment revealed that ROR1 maintains the key amino acid residues in conserved regions of protein kinase domains, including the VAIK motif, catalytic loop, and activation segment. The major difference was the substitution of glycine by cysteine in the glycine loop (**Figure 3**). Interestingly, the same glycine residue in the glycine loop was substituted by asparagine in ROR2. Since the kinase activity of ROR2 has been confirmed by multiple studies (14, 55, 69), it is likely that the glycine by cysteine substitution may not abolish the kinase activity. It has been demonstrated that Wnt5a induced homodimerization and autophosphorylation of ROR2. It is conceivable that activation of ROR1 kinase activity also requires engagement with its ligand such as Wnt5a. Multiple factors could contribute to the failure to detect the tyrosine kinase activities of ROR1. Systematic biochemical studies are needed to uncover the enzymatic properties of ROR1, including transphosphorylation, autophosphorylation, and phosphorylation sites, as well as substrate preference interactions of proteins and nucleotides.

## Therapeutic Strategies Targeting ROR1 in Malignancies

To date, several therapeutic strategies against ROR1 have been developed and evaluated in clinical trials (**Table 1**) and preclinical studies (**Table 2**). Most targeted cancer therapies use either small-molecule drugs or mAbs-based strategies (74, 75). mAbs block ligand binding directly and activate the immune system to eliminate tumor cells (76). Small-molecule tyrosine kinase inhibitors (TKIs) are ATP-competitive inhibitors that target the catalytic domains in tyrosine kinases (77). Currently, multiple mAbs against ROR1 have been developed (78–83). These mAbs mediated antibody-dependent cellular cytotoxicity (ADCC), complement-dependent cytotoxicity (CDC), internalization of ROR1, and apoptosis in malignant cells expressing ROR1. To date, cirmtuzumab is the only monoclonal antibody (mAb) targeting ROR1 that has been evaluated in clinical trials. A phase I trial of cirmtuzumab in patients with CLL has been finished with beneficial effects observed in patients (84). A phase Ib and phase I/II trials on cirmtuzumab combined with paclitaxel or ibrutinib in patients with breast cancer or CLL/MCL are currently ongoing.

**Table 1:** ROR1-targetd therapies in clinical trials.

Drug	Description	Disease	ClinicalTrials.gov Identifier
Cirmtuzumab	mAb	CLL MCL Breast cancer	NCT03088878 NCT02776917 NCT02222688 NCT02860676
VLS-101	ADC (MMAE)	Lung cancer Breast cancer Hematological neoplasms	NCT03833180 NCT04504916
NBE-002	ADC (PNU-159682)	Triple Negative Breast Cancer Advanced Solid Tumor	NCT04441099
NVG-111	Bispecific antibody to ROR1 and CD3	CLL MCL	NCT04763083
CAR-T	CAR-T	Lung cancer Breast cancer Hematological neoplasms	NCT02706392

ADC, antibody drug conjugate; CLL, chronic lymphocytic leukemia; mAb, monoclonal antibody; MCL, mantle cell lymphoma; MMAE, Monomethyl auristatin E.

**Table 2:** ROR1-targeted drugs in preclinical studies.

Drug	Description	Disease	Reference
KAN0441571C	Inhibitor	DLBCL	(64)
KAN0439834	Inhibitor	CLL Pancreatic cancer	(62, 63)
Strictinin	Inhibitor	Breast cancer	(70)
ARI-1	Inhibitor	Lung cancer	(71)
2A2-OSU-2S-ILP	Immunonanoparticle with OSU-2S	CLL MCL	(72, 73)

CLL, chronic lymphocytic leukemia; DLBCL, diffuse large B cell lymphoma; MCL, mantle cell lymphoma.

Based on ROR1-targeted mAbs, antibody drug conjugate (ADC), bispecific T cell engager (BiTE), and chimeric antigen receptor (CAR) T cells have also been developed, some of which are currently being evaluated in clinical trials. VLS−101 is an ADC comprising cirmtuzumab and monomethyl auristatin E (MMAE), an agent that inhibits cell division by blocking the polymerization of tubulin. It is effective to target ROR1-positive malignant cells by binding to ROR1 that leads to rapid internalization of MMAE to induce cell death (85). Similarly, NBE-002 is an ADC that consists of a humanized mAb against ROR1 that is conjugated to a derivative of the highly potent anthracycline PNU-159682, which shows significant anti-tumor activity in patient-derived xenograft models of breast cancer, lung adenocarcinoma, ovarian carcinoma, and sarcomas (86). Clinical trials evaluating VLS−101 and NBE-002 in hematological malignancies and solid tumors are ongoing. NVG-111 is a first-in-class humanized ROR1 and CD3 BiTE that utilizes the inherent cytotoxic potential of resident T cells to target ROR1-positive malignant cells. Preclinical studies showed promising T-cell-mediated cytotoxicity of NVG-111 in CLL and solid tumors (87). The Clinical trial of NVG-111 in CLL and MCL is active now. CAR-T cells targeting ROR1 also demonstrate strong potency and specificity to malignant cells expressing ROR1, which has been evaluated in a clinical trial in patients with ROR1-positive hematological and solid malignancies (28). Furthermore, the immunonanoparticle-mediated ROR1-targeted delivery of OSU-2S, a sphingosine analogue with anti-tumor activity, showed promising potency in ROR1-positive malignant cells in preclinical studies. Subsequent large-scale clinical trials are highly warranted (72, 73).

Since the kinase activity of ROR1 has not been clearly defined, little effort has been made to identify small-molecule inhibitors of ROR1. Nonetheless, a small molecule compound dubbed KAN0439834 was found to inhibit the survival of CLL and pancreatic cancer cells, as well as phosphorylation of ROR1 in cell-based assays (62, 63). The potency of KAN0439834 to induce apoptosis in CLL cells was as strong as that of venetoclax, a BCL-2 inhibitor approved for treating patients with CLL in *in vitro* assays. KAN0439834 seems to target the tyrosine kinase domain of ROR1 and inhibit Wnt5a-induced phosphorylation of ROR1, which leads to the deactivation of downstream signaling proteins SRC, AKT, PKC, and MAPK. In pancreatic cancer cells, the efficiency of KAN0439834 can be enhanced by erlotinib and ibrutinib, a small molecule inhibitor of EGFR and BTK, respectively. Based on this compound, a second-generation small molecule ROR1 inhibitor KAN0441571C with improved potency and pharmacokinetics to inhibit DLBCL in a zebrafish model was developed by the same group (64). KAN0441571C and venetoclax showed a promising combination effect to eliminate DLBCL cells *in vitro*. Although these compounds are effective in decreasing the phosphorylation of ROR1 and c-Src in malignant cells, the mechanism of action requires further investigation by performing biochemical assays using isolated proteins. Interestingly, another study identified a small molecule designated ARI-1 that targeted the extracellular CRD of ROR1, thereby blocking Wnt5a binding (71). ARI-1 was shown to suppress non-small cell lung cancer cell growth *in vitro* and *in vivo* (71). Strictinin, a compound isolated from *Myrothamnus flabellifolius* was also reported to bind to the intracellular domain of ROR1, which inhibited AKT phosphorylation and survival of breast cancer cells (70). Further investigations are needed to characterize the mechanism of action of these compounds before clinical trials.

Considering the promising outcomes of anti-ROR1 immunotherapies, we believe that small molecule inhibitors targeting ROR1 should be actively pursued by performing cell-based biochemical screening. A preferred cell-based screening system should contain cells whose survival and proliferation depend on the hyperactivation or overexpression of ROR1. The inhibitors thus identified may target ROR1 directly or act indirectly on other signaling components downstream of ROR1. To identify inhibitors targeting the kinase domain of ROR1, a kinase assay system needs to be established with appropriate substates and effective detection methods. To date, the majority of tyrosine kinase inhibitors bind to catalytic centers and block the binding of ATP.

## Conclusions

Receptor tyrosine kinase ROR1 is an excellent target for the development of therapeutic drugs to treat CLL and several types of solid tumors. Major progress has been made in developing antibody-based immunotherapies targeting ROR1 in preclinical and clinical studies. The identification of small-molecule compounds targeting ROR1 still lags behind due to a poor understanding of ROR1 kinase activity. We believe that ROR1 should have intrinsic kinase activities, but further studies are required to understand its enzymatic properties. While cell-based screening systems should be employed to find inhibitors targeting ROR1 directly or indirectly, biochemical assays are required to identify inhibitors targeting its kinase domain.

## Author Contributions

(I) Conception and design: YC, XX, and ZZ. (II) Administrative support: None. (III) Provision of study materials or patients: None. (IV) Collection and assembly of data: None. (V) Data analysis and interpretation: None. (VI) Manuscript writing: All authors. (VII) Final approval of manuscript: All authors. All authors contributed to the article and approved the submitted version.

## Funding

We thank 100 Top Talents Program of Sun Yat-sen University,National Natural Science Foundation of China (NSFC, Grant No. 31871400), Shenzhen Science and Technology Innovation Commission (JCYJ20190809172403604) and Sanming Project of Medicine in Shenzhen (No. SZSM201911004) for supporting the manuscript preparation and publication.

## Conflict of Interest

The authors declare that the research was conducted in the absence of any commercial or financial relationships that could be construed as a potential conflict of interest.
